# Inhibition of TDP-43 Aggregation by Nucleic Acid Binding

**DOI:** 10.1371/journal.pone.0064002

**Published:** 2013-05-30

**Authors:** Yi-Chen Huang, Ku-Feng Lin, Ruei-Yu He, Pang-Hsien Tu, Jiri Koubek, Yin-Chih Hsu, Joseph Jen-Tse Huang

**Affiliations:** 1 Graduate Institute of Life Sciences, National Defense Medical Center, Neihu, Taipei, Taiwan; 2 Institute of Chemistry, Academia Sinica, Nankang, Taipei, Taiwan; 3 Institute of Biomedical Sciences, Academia Sinica, Nankang, Taipei, Taiwan; Consejo Superior de Investigaciones Cientificas, Spain

## Abstract

The aggregation of TAR DNA-binding protein (TDP-43) has been shown as a hallmark of amyotrophic lateral sclerosis (ALS) and frontotemporal lobar degeneration (FTLD) since 2006. While evidence has suggested that mutation or truncation in TDP-43 influences its aggregation process, nevertheless, the correlation between the TDP-43 aggregation propensity and its binding substrates has not been fully established in TDP-43 proteinopathy. To address this question, we have established a platform based on the *in vitro* protein expression system to evaluate the solubility change of TDP-43 in response to factors such as nucleotide binding and temperature. Our results suggest that the solubility of TDP-43 is largely influenced by its cognate single-strand DNA (ssDNA) or RNA (ssRNA) rather than hnRNP, which is known to associate with TDP-43 C-terminus. The direct interaction between the refolded TDP-43, purified from *E.coli*, and ssDNA were further characterized by Circular Dichroism (CD) as well as turbidity and filter binding assay. In addition, ssDNA or ssRNA failed to prevent the aggregation of the F147L/F149L double mutant or truncated TDP-43 (TDP_208–414_). Consistently, these two mutants form aggregates, in contrast with the wild-type TDP-43, when expressed in *Neuro2a* cells. Our results demonstrate an intimate relationship between the solubility of TDP-43 and its DNA or RNA binding affinity, which may shed light on the role of TDP-43 in ALS and FTLD.

## Introduction

The 43 kDa TAR DNA-binding protein (TDP-43) and its C-terminal proteolytic fragments have been identified as the major component of inclusion bodies in amyotrophic lateral sclerosis (ALS) and frontotemporal lobar degeneration with unbiquitin-positive inclusions (FTLD-U) [1], [2], [3], [4], [5]. Recently, it has also been recognized as a histopathological marker of Alzheimer’s [6], Parkinson’s [7], as well as Huntington’s diseases [8]. TDP-43 is a multifunctional protein involved in mRNA splicing, degradation, stabilization, translation and transportation [9], [10], [11], [12]. It is located mainly in the nucleus but appears to shuttle between the nucleus and cytosol [11], [13], [14]. Under pathological conditions, hyper-phosphorylated, ubiquitinated, and N-terminally truncated TDP-43 species are often found in cytosolic inclusions in patients [14], [15], [16].

TDP-43 contains two highly conserved RNA-recognition domains, RRM1 and RRM2, which are involved in DNA or RNA binding, preferentially with sequence enriched in UG or TG dinucleotide repeats [17], [18], [19]. These RRM domains are followed by a glycine-rich C-terminal tail which is known to interact with different isoforms of the heterogeneous nuclear ribonucleoprotein (i.e. hnRNP A1 and A2/B1) [9], [17], [20], [21], [22], [23], [24] that is involved in the cystic fibrosis transmembrane conductance regulator (CFTR) exon skipping [17], [18]. The C-terminus of TDP-43 is known to control its cellular location as well as its solubility; fragments derived from this region have also implicated this region as determinants of the aggregation process [4], [24], [25], [26], [27], [28]. In addition, it has been demonstrated that certain mutations in the TDP-43 C-terminus accelerate its aggregation and increase toxicity in *in vivo* and *in vitro* studies [11], [29].

Recently, it has been shown that the disruption of DNA or RNA binding through mutation or RRM1 truncation may alter the solubility and localization of TDP-43. Nuclear bodies or aggregates appear in the nucleus when DNA binding ability is abolished or after the treatment of the nucleus with RNAse A [17], [22], [30]. Moreover, the TDP-43 C-terminal fragment (TDP_182–414_), which contains only RRM2 and glycine-rich domains, remains soluble in a cellular environment [3], [15] but forms inclusion-like foci upon treatment of the cells with RNAse A [3]. These results have suggested that the aggregation of TDP-43 is influenced by DNA/RNA binding and the cognate DNA or RNA might prevent TDP-43 from transforming into oligomers or large aggregates. On the other hand, others have suggested that RNA might stabilize or convert the native TDP-43 into a misfolded conformation that is toxic [31], [32]. In fact, it has been demonstrated that the participation of nucleic acids may affect the aggregation of other proteins involved in different protein misfolding diseases. They may either serve as an inducer [33], [34], [35], [36], [37], [38] or an inhibitor in the amylodogenesis or aggregation process [39], [40]. Clearly, there is a need to sort out these different viewpoints before we can begin to understand the TDP-43 aggregation process as well as its proteinopathy. Towards this end, we have embarked on studies directed toward clarifying the role of DNA or RNA, the major binding partner involved in transcription, translation, and gene regulation within the cell, on the complex TDP-43 aggregation process.

It is well known that bacterially expressed recombinant TDP-43 is intrinsically aggregation-prone and may form fibrillar aggregates [25], [26], which may impede us in the proposed single-strand DNA (ssDNA) or RNA (ssRNA) binding experiments. To simplify the study, we have established a platform based on rabbit reticulocyte cell-free system to express soluble TDP-43 and screen the factors that might impact the TDP-43 aggregation process [41]. Different factors, including the incubation temperatures [4], [42], incubation time, DNA/RNA and the length and concentration of the oligonucleotides, are compared. The direct influence of oligonucleotides on the TDP-43 aggregation is monitored by turbidity assay with purified *E.coli* TDP-43 after refolding in the presence and absence of ssDNA. To further elucidate the implication of the DNA or RNA binding effects within cell, we have also transiently expressed truncated (TDP_208–414_) and double mutants of TDP-43 (F147L/F149L) in *Neuro2a* cells, a mouse neuroblastoma cell line. Our results have provided new evidence that the aggregation process of TDP-43 is not only influenced by C-terminal fragment of the protein but also by its binding partners. These underscore an important consideration that should be included in the design of in pathologic and therapeutic studies against ALS and FTLD.

## Materials and Methods

### Plasmid Constructions

The DNA sequence encoding human TDP-43 was introduced into several plasmids for different applications. For cell transfections and cell-free reactions, the genes of TDP-43 and TDP_208–414_ including the additional encoded FLAG-tag at the 5′-end, were ligated between the XhoI-NotI restriction enzyme sites in the pCMVTNT vector (Promega, USA) and the NcoI-XhoI enzyme sites in pIVEX2.3d vector (Roche, Germany), respectively. For TDP-43 expression in *E. coli*, pET17b plasmid was used. All the constructs were sequenced by Tri-I Biotech, Inc. (Taiwan) and the inserted sequence of the recombinant TDP-43 was verified.

### The Effects of Oligonucleotide Binding on the Aggregation of the TDP-43 Protein in Cell-free Systems

The eukaryotic cell-free system derived from the rabbit reticulocyte lysate (TNT T7 Quick-Coupled Transcription/Translation System, Promega, USA) and the prokaryotic cell-free system derived from the *E.coli* extract (S30 T7 High-Yield protein expression system Promega, USA) were applied to generate the TDP-43 proteins and provide a cellular mimicking environment to study the TDP-43 protein aggregation. After the synthesis was completed at 30°C for 2 h, the cell-free reactions were further incubated at either 30 or 37°C (1–4 h) for the formation of the TDP-43 aggregates. To evaluate the influence of ssDNA/RNA, various oligonucleotides [(TG), (UG) or (CA)] repeated sequences with 6 to 24 nt lengths and 4–10 µM concentrations were added into the cell-free reaction immediately after the synthesis. At different time points after incubation, the samples were divided into supernatant and pellet fractions by centrifugation at 16,100×*g* for 30 minutes at 4°C. The pellet was then dissolved by SDS-PAGE loading buffer (50 mM Tris-HCl pH 6.8, 7.5% glycerol, 1% SDS, 0.02% bromophenol blue and 1% β-mercaptoethanol) and the proteins in the supernatant were precipitated by acetone followed by re-suspension with the SDS-PAGE loading buffer. The TDP-43 in both fractions was detected by western blotting with a primary anti-FLAG M2 monoclonal antibody (Sigma, USA) and a secondary anti-mouse antibody conjugated with HRP (Sigma, USA). The antibodies-TDP-43 complexes were detected on Biospectrum® AC image system (UVP, USA) using a chemiluminescent substrate for HRP (Millipore, USA). The blotting signals were analyzed by Image J (National Institutes of Health, Bethesda, MD) and the fractions of TDP-43 protein in supernatant were obtained by the equation S/(S+P), where S and P denote the detected signals of the TDP-43 in the supernatant and pellet fractions, respectively. All the experiments for each sample were repeated three times.

### Circular Dicroism Measurement of TDP-43

The native and refolded TDP-43 as well as mutants (2–3 µM) were dissolved in phosphate buffer and incubated at room temperature. The CD spectra of the resulting protein solutions were recorded in 1 mm quartz cuvette on the J-815 CD spectrometer (JASCO, Japan). All data were collected from 200 to 260 nm with the scanning speed of 100 nm/min. Three scans were averaged for each sample.

### Detection of TDP-43 Protein Aggregates by Turbidity Assay and Filter Binding Assay

The refolded TDP-43 proteins (3 µM) in the absence or presence of different oligonucleotides [1 µM of (CA)_6_ or (TG)_6_] were incubated in refolding buffer at 25°C with a continuous agitation (1400 rpm) in the thermomixer (Eppendorf, Germany). At different time points after incubation, the turbidity of TDP-43 proteins was detected by measuring the absorbance at 600 nm in a UV-spectrophotometer (Beckman Coulter) at room temperature. The filter binding assay was followed with the published protocols [25]. 0.1 µM of (TG)_12_ was added into the protein solution with different concentration (0.5, 1.0, and 2.0 µM) at 37°C. After 1 h, the mixture was loaded onto the nitrocellulose membrane (GE healthcare, American) presoaked with buffer containing 0.4 M KOH, rinsed with water, and washed with diluted buffer (100 mM NaCl, 5 mM EDTA, 5 mM DTT, and 10% glycerol, pH 8.0). The fluorescence signal on the membrane was obtained from the Typhoon TRIO imager (GE healthcare).

## Results

### Aggregation Propensity of TDP-43 Differs in Different Cell-free Systems

**Figure 1:**
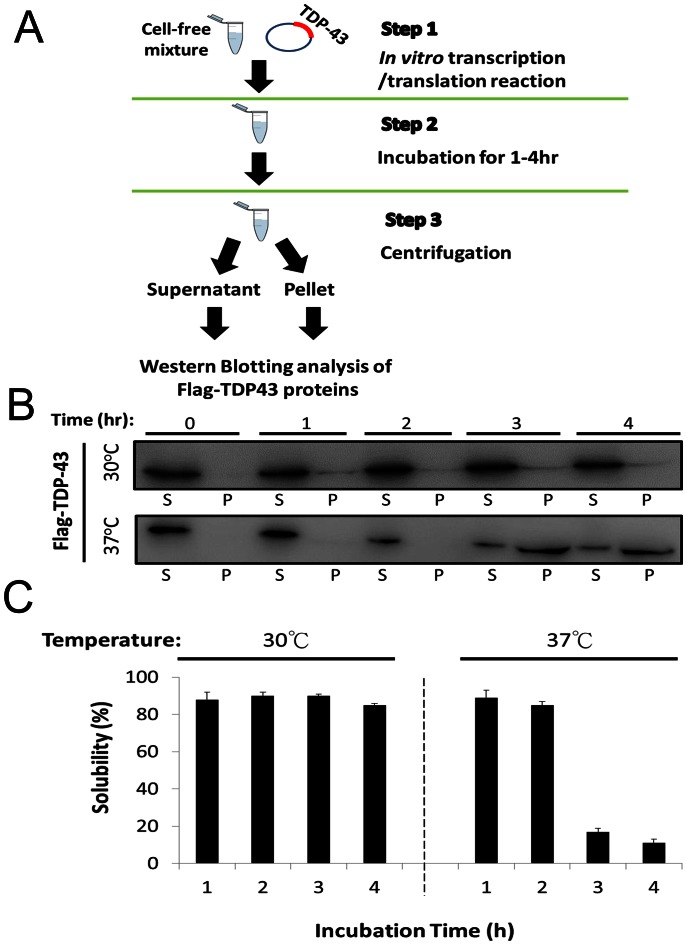
The solubility of human TDP-43 in rabbit reticulocyte cell-free system under different incubation conditions. (**A**) A flow chart of solubility determination consisting of protein synthesis (step 1), time-course incubation (step 2), and the quantification of TDP-43 in different fractions (step 3). (**B**) TDP-43 in supernatant (S) and pellet (P) fractions are visualized by western blotting after SDS-PAGE under different incubation time (1–4 h) and temperature (30°C and 37°C) (step 2). (**C**) The solubility of TDP-43 was calculated by quantifying the gel bands of S and P in (**B**) with Image J [51] and the formula, Solubility (%) = S/(S+P)*100.

In this study, we have employed two different cell-free systems, derived from *E. coli* (prokaryotes) [43] or rabbit reticulocyte (eukaryotes), to produce TDP-43 proteins in a cellular-like environment (step 1, *In vitro* transcription/translation reaction). The generated TDP-43 or its derivatives are further incubated in these systems to evaluate the temporal dependence of their solubility at different temperatures in the presence or absence of its cognate oligonucleotides (step 2, Incubation) by quantifying the TDP-43 proteins in the supernatant and pellet fractions of each system with western blotting (step 3, Centrifugation and quantification) (Figure 1A).

Interestingly, the TDP-43 generated from different cell-free systems substantially differs in their aggregating potencies. Only the eukaryotic system provides soluble TDP-43 proteins for examination of the influence of DNA on the TDP-43 aggregation process. In the *E. coli* cell-free system, the TDP-43 generated at various temperatures (from 16 to 37°C) is mostly found in the pellet fraction (Figure S1A). This outcome suggests that the recombinant TDP-43 in this system has an extremely high aggregating propensity, and thus, forms aggregates during synthesis. The solubility of the obtained TDP-43 is not affected by co-incubating concentrated DNA with the aggregates at different temperatures (Figure S1B). In contrast, most of the TDP-43 generated from rabbit reticulocyte cell-free system resides in the supernatant fraction at 30°C (solubility ∼90%) (Figure 1B and C) with it’s maximum protein expression level within 2 h (Figure S2). After its synthesis, the expressed TDP-43 protein could remain soluble for at least 4 h at 30°C (Figure 1B, upper panel) or 2 h at 37°C (Figure. 1B, lower panel) and reach the concentration of 1 µM (Figure S3). Upon incubation at 37°C, the TDP-43 in the soluble fraction decreases from 80 to 20% after 3 h and this trend continues after an additional hour of incubation (∼10% soluble). This dramatic change in the TDP-43 solubility during incubation at 37°C for 3 h in the cell-free system provides an excellent experimental condition to examine the impact of DNA or RNA on the TDP-43 aggregation process. In other words, we have taken advantage of this non-equilibrium system to reveal some thermodynamic properties of the TDP-43 aggregation. It is worth to note that the TDP-43 from *E.coli* cell free system has lower solubility compare to those from the rabbit reticulocyte cell-free system. This may result from the higher translation rate/yield in the *E.coli* cell-free system (Figure S3B) or the different components in these two systems.

### The Presence of Cognate DNA and RNA Diminished Aggregation of TDP-43 in Rabbit Reticulocyte Cell-free System

**Figure 2:**
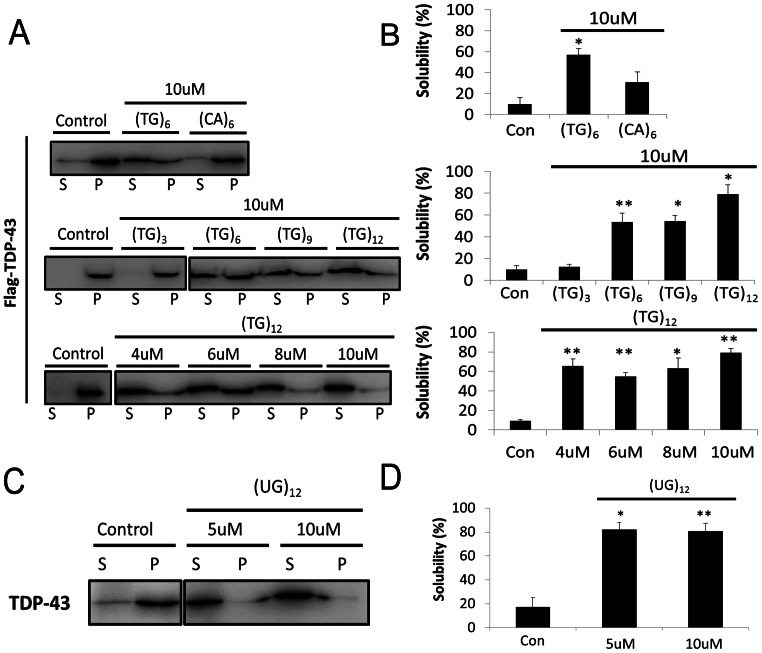
The effects of ssDNA and ssRNA on TDP-43 solubility. (**A**) The supernatant (S) and pellet (P) of TDP-43, incubated at 37°C for 3 h, in the absence (control) or presence of various ssDNA [i.e. (CA)_6_, (TG)_3_, (TG)_6_, (TG)_9_, and (TG)_12_] with different concentration (4–10 µM) were analyzed by western blotting. (**B**) The solubility of TDP-43 in the absence or presence of various ssDNA with different concentration was derived from the intensity of S and P in (**A**), and the P-values were calculated by Student’s t-test (^**^, P<0.01 and ^*^, P<0.05) (**C**) The gel analysis of the TDP-43 supernatant (S) and pellet (P) incubated with 5 or 10 µM of ssRNA, (UG)_12_, for 3 h at 37°C with anti-Flag antibody. (**D**) The percentage of S and P was obtained from quantification of the intensity in (**C**) by using Image J [51].

TDP-43 has been reported to be a nucleic acid binding protein that prefers (TG or UG)-rich sequences [9], [17], [22], [44]. To study the impact of DNA binding in the aggregation of TDP-43, two ssDNAs bearing different nucleotide sequences, namely (TG)_6_ and (CA)_6_, were co-incubated with newly synthesized protein in the cell-free reaction mixture for 3 h at 37°C. Intriguingly, the results showed the DNA significantly prevents the TDP-43 from aggregation. The solubility of TDP-43 in the presence of (TG)_6_ and (CA)_6_ were around 60 and 30%, respectively, which were much higher than that of the control (∼10%) in the absence of exogenous ssDNA (Figure. 2A and B, upper panel).

Since the (TG)_6_ is reported to have a much higher binding affinity for TDP-43 than (CA)_6_ [9], [17], we surmise that the stronger effect of (TG)_6_ on TDP-43 solubility is correlated with its stronger binding to TDP-43. To test this hypothesis, the influence of different length of (TG)_n_ repeats (n = 3, 6, 9, and 12) oligonucleotides on the solubility of TDP-43 have been characterized and compared. According to the literature, TDP-43 could only bind to longer (TG)_n_ repeats (n≧−6) and its affinity to DNA increases with the n value [17]. As expected, the (TG)_12_ fragment inhibits most of TDP-43 from aggregation, maintaining the solubility of the TDP-43 at ∼80%, whereas the solubility of TDP-43 (∼50%) is affected by (TG)_6_ or (TG)_9_ to a lesser extent (Figure 2A and B, middle panel). In contrast, (TG)_3_ has a negligible effect on the solubility compared with the control (solubility: ∼10%) (Figure 2A and B, middle panel). Moreover, we find that the concentration of external ssDNA (4 µM) is sufficient for DNA binding in these experiments, since the solubility of TDP-43 is similar (60–80%) in the presence of 4 to 10 µM of (TG)_12_ (Figure. 2B, lower panel). Therefore, these results demonstrate that the reduction in TDP-43 aggregation is highly correlated with the binding affinity between ssDNA and TDP-43.

In addition to ssDNA, ssRNA could similarly reduce TDP-43 aggregation during co-incubation. In the presence of 5 and 10 µM of (UG)_12_, the fraction of the TDP-43 protein in the supernatant was raised from ∼15% to ∼80% after the incubation at 37°C for 3 h (Figure. 2C and D).

### Mutation/Truncation Reduces TDP-43 Solubility by Suppressing Competitive Binding of Oligonucleotides

**Figure 3:**
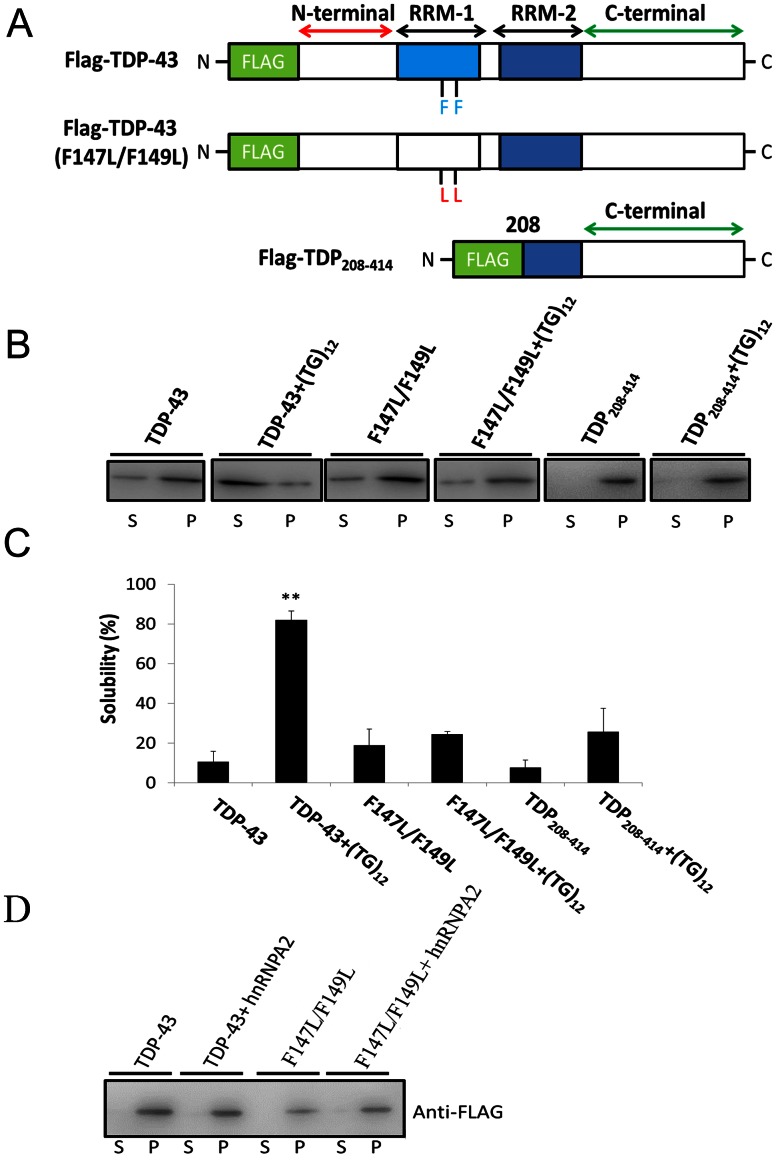
The solubility of wild-type, mutant (F147L/F149L), and N-terminally truncated (TDP_208–414_) TDP-43 in the absence or presence of ssDNA or ssRNA. (**A**) The schematics of wild-type and mutant (F147L/F149L) TDP-43. The DNA/RNA binding domains, RRM1 and RRM2, were highlighted in light and dark blue, respectively. The N-terminus FLAG tag (green) was designed for the recognition during western blotting. (**B**) The western blot analysis of the wild-type and mutant (F147L/F149L) TDP-43 under the incubation with 10 µM (TG)_12_ or (UG)_12_ for 3 h at 37°C with anti-Flag antibody. (**C**) The solubility of TDP-43 was derived from the band of S and P in (**B**). (D) The solubility of wild-type and mutation TDP-43 in the presence and absence of hnRNP A2.

It has been reported that the two RRM domains of TDP-43 are the major binding sites of oligonucleotides (Figure 3A) [9], [22], [44]. Recently, it has been reported that two mutations (F147L/F149L) on the RRM1 domain significantly reduce the nucleotide binding ability of TDP-43 [17]. In fact, the aromatic cores of phenylalanine residues may stack with the bases in oligonucleotides upon binding and therefore stabilize the TDP-43/nucleotides interactions. Apparently, the F147L/F149L mutations abolish these interactions, thereby destabilizing the TDP-43/oligonucleotide complexes [17]. The inability of F147L/149L to bind oligonucleotides decreases the effectiveness of cognate DNA in maintaining the TDP-43 solubility in the rabbit reticulocyte cell-free system. Only about 20% of the F147L/149L remains in the soluble fraction in the presence of (TG)_12_ (Figure 3 B and C), which is significantly different from the wild type TDP-43 in the presence of same oligonucleotides (∼80%) after incubation at 37°C for 3 h.

It has long been discussed that the truncated TDP-43 forms (25 kDa and 35 kDa) are directly related to TDP-43 proteinopathy and may result in specific protein aggregation [28], [41]. Here, we have confirmed the solubility change in the presence of truncated TDP_208–414_, the 25 kDa C-terminal fragment in human disease, in the presence and absence of ssDNA. Moreover, our results reveal that (TG)_12_ is not able to prevent TDP-43 from aggregation; the solubility is only slightly increased (∼ 25%) after incubating with (TG)_12_ for 3 h compared with the control (∼ 8%) (Figure 3 B and C). Since the truncated TDP-43 is lacking the N-terminus, RRM1, and possible part of RRM2, it is speculated that they may largely lose their DNA/RNA binding ability. As we are proposing, the binding with oligonucleotides is a critical requirement for DNA/RNA in preventing TDP-43 aggregation.

Interestingly, hnRNP has been known to associate with theTDP-43 C-terminus to regular the exon 9 splicing [21]. Here, we have also try to examine the solubility change of TDP-43 in the presence of human hnRNP A2 expressed and purified from *E.coli*. Different from the case in ssDNA and ssRNA, the presence of hnRNP A2 was not able to increase the solubility of TDP-43 or its mutant (F147L/F149L) (Figure 3D).

### The Inhibition of Purified *E. coli* TDP-43 Aggregation by ssDNA

**Figure 4:**
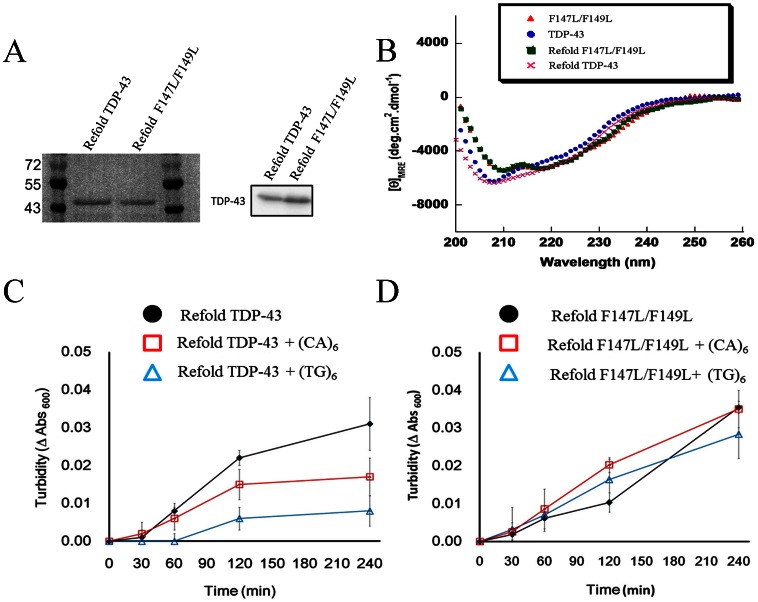
Characterization of the refolded TDP-43 protein. (**A**) The refolded wild-type and double-mutation (F147L/F149L) TDP-43 identified with coomassie blue in the SDS-PAGE, or by western blotting with TDP-43 antibody. (**B**) The secondary structure of refolding and native TDP-43 characterized by CD spectroscopy. The aggregation of the TDP-43 (**C**) and its mutant (F147L/F149L) (**D**) in the absence or presence of oligonucleotides incubated at 25°C with a continuous agitation from 0 to 4 h was monitored by the solution turbidity at 600 nm. The averaged results ± std are presented for protein alone (•), or incubated with (CA)_6_ (**□**), and (TG)_6_ (**▵**).

To confirm the direct interaction between ssDNA and TDP-43 and rule out the possibility that the endogenous proteins/DNAs within the cell free system that may diminish the TDP-43 aggregation, we have also conducted similar experiments with purified proteins expressed from *E. coli.* As mentioned earlier, the overexpression of recombinant TDP-43 proteins in *E. coli* leads to the formation of insoluble inclusion bodies and most of these proteins are found in aggregates at 37°C [25], [26]. To produce soluble TDP-43, the protein is expressed at low temperature and lysed under the presence of detergent and glycerol (Methods S1). Unfortunately, the expressed native protein has very low yield and was only able to remain soluble within 30 mins, which is not suitable for both turbidity and DNA binding assay. Hence, we try to obtain refolded TDP-43 by re-dissolving of the aggregates with 6 M urea followed by a series of dialysis against refolding buffer (Methods S1). These proteins have high purity as confirmed by SDS-PAGE together with western blotting (Figure 4A) and did not remain aggregated in the stacking gel (data not shown). The secondary structure of refolded protein (TDP-43 and F147L/F149L) is quickly compared with fresh native ones by CD spectroscopy (Figure 4B) to ensure they have similar structure.

The binding of the ssDNA on wild type/mutant TDP-43 was confirmed by the filter binding assay with 6-carboxyfluorescein (FAM)-labelled (TG)_12_ and (CA)_12_. As expected, the refolded TDP-43 exhibited much stronger binding ability toward (FAM)-(TG)_12_ than mutant (F147L/F149L) (Figure S4). We had also characterized the binding activity of refolded TDP-43 with (FAM)-labelled (TG)_12_ and (CA)_12_ by electrophoretic mobility shift assay (EMSA) (data not shown) and confirmed the direct interaction between recombinant protein and nucleic acid. The aggregation process is monitored by measuring the solution turbidity of the refolded TDP-43 proteins in the presence or absence of ssDNA at different time points within 4 h. As shown in Figure 4 C and D, the refolded proteins gradually form aggregates and the corresponding measured turbidity increases with time. Consistently, the aggregation rate of TDP-43 could be influenced by co-incubating the protein with ssDNAs [i.e. (TG)_6_ and (CA)_6_]. Comparing with the TDP-43 in the absence of ssDNA, the lower turbidity of TDP-43 with (CA)_6_ suggests that the size of aggregates may be efficiently reduced and this effect is even more stronger in the case of (TG)_6_ (Figure 4C). The observed lag phase preceding the TDP-43 aggregation might indicate a nucleation-dependent polymerization process, which is longer in TDP-43 with (TG)_6_ (>60 min) than with (CA)_6_ (<30 min) or control. On the contrary, co-incubation of ssDNAs with the purified mutant TDP-43 (F147L/149L) had no significant effect on the protein aggregation process (Figure 4D). The curves of turbidity change of the mutant TDP-43 (F147L/F149L) in the presence of (TG)_6_/(CA)_6_ are similar to those without exogenous DNAs (Figure 4D).

In an attempt to characterize the morphology of the refolded TDP-43 in the presence and absence of single-strand oligonucleotides, we had observed the TDP-43 structure by electron microscopy (EM) with secondary antibody conjugated on gold nanoparticles. Instead of fiber-like aggregates, only amorphous and/or irregular aggregates could be found in our refolded TDP-43 (Figure S5). However, due to the high inhomogeneity of the solution, the morphological change in TDP-43 aggregates before/after the addition of ssDNA was not further discussed.

### Different Distributions of Wild Type, Mutant, and Truncated TDP-43 in the *Neuro2a* Cells

**Figure 5:**
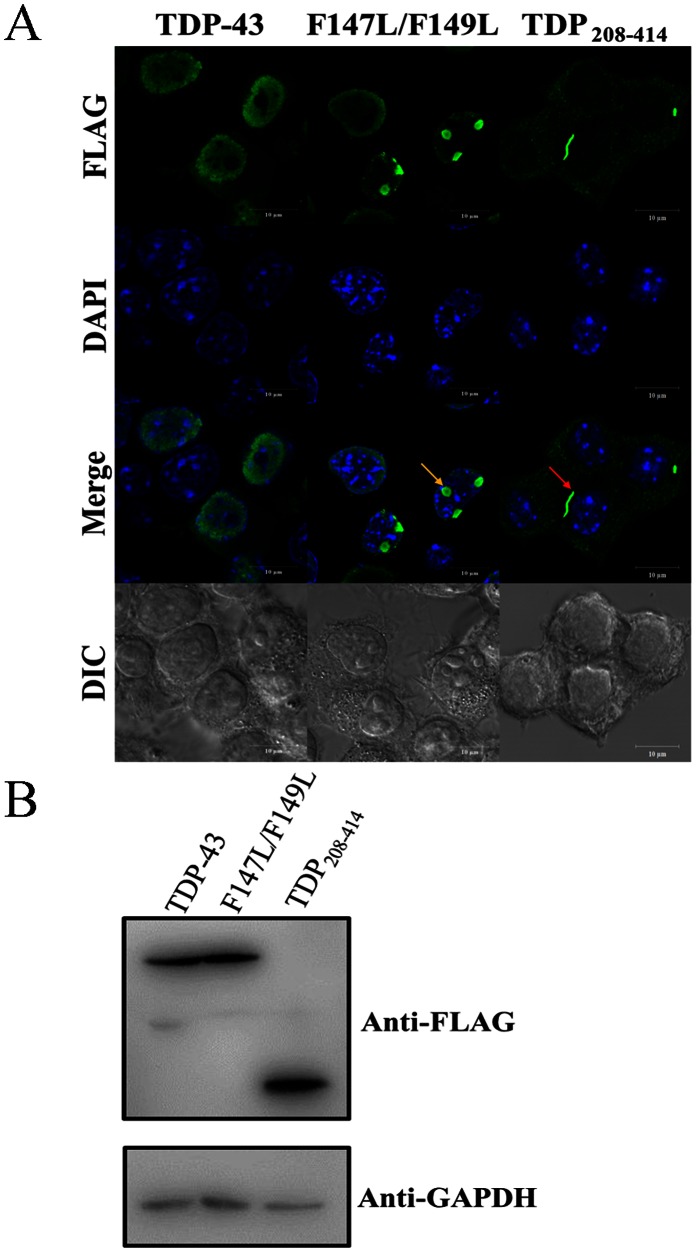
Confocal immunofluorescent images of *Neuro2a* cells of TDP-43 variants. (**A**) Inclusions were observed only in the mutant (F147L/F149L, indicated by yellow arrow) and truncated (TDP_208–414_, indicated by red arrow) TDP-43 rather than the wild-type protein in the cell. Secondary anti-mouse antibody conjugated with Alexa 488 reacted with primary anti-FLAG antibodies hybridized with FLAG-tagged TDP-43 variants. DAPI was used for visualizing nuclei. (Scale bar = 10 µm). (**B**) The expression level for wild type, mutant (F147L/F149L), and truncated (TDP_208–414_) TDP-43 on the SDS-PAGE with western blotting. GAPDH is served as loading controls for each lane.

Finally, we have determined whether or not the binding of endogenous oligonucleotides may influence the solubility and aggregation process of TDP-43 in the neuron cell model by monitoring the distributions of FLAG-tagged wild type, mutant (F147L/F149L), and N-terminally truncated (TDP_208–414_) TDP-43 in the *Neuro2a* cells (Figure 5A) (Methods S1). By applying immunofluorescent staining techniques coupled with confocal microscopy, we observe highly fluorescent spots, which indicate the presence of aggregates in the cells transfected with the double mutant (F147L/F149L) and N-terminally truncated protein (TDP_208–414_), but not wild type TDP-43. Interestingly, most of the aggregates of the mutant F147L/F149L are found to be round in shape (∼2–3 µm in diameter, pointed by yellow arrow) in the nucleus rather than in the cytosol, suggesting that the presence of nuclear localization sequence (NLS) results in the location of this mutant in the nucleus, as expected, and the loss of DNA/RNA binding of this mutant effectuated aggregates. On the other hand, the truncated TDP_208–414_, which did not contain the NLS sequence, has failed to enter through the nucleus membrane and formed linear aggregates (∼1 µm in width, ∼2–8 µm in length, pointed by red arrow) in the cytosol. The expression level for wild type, mutant (F147L/F149L), and truncated (TDP_208–414_) TDP-43 are similar in the *Neuro2a* cells as shown on the SDS-PAGE with western blotting (Figure 5B).

## Discussion

**Figure 6:**
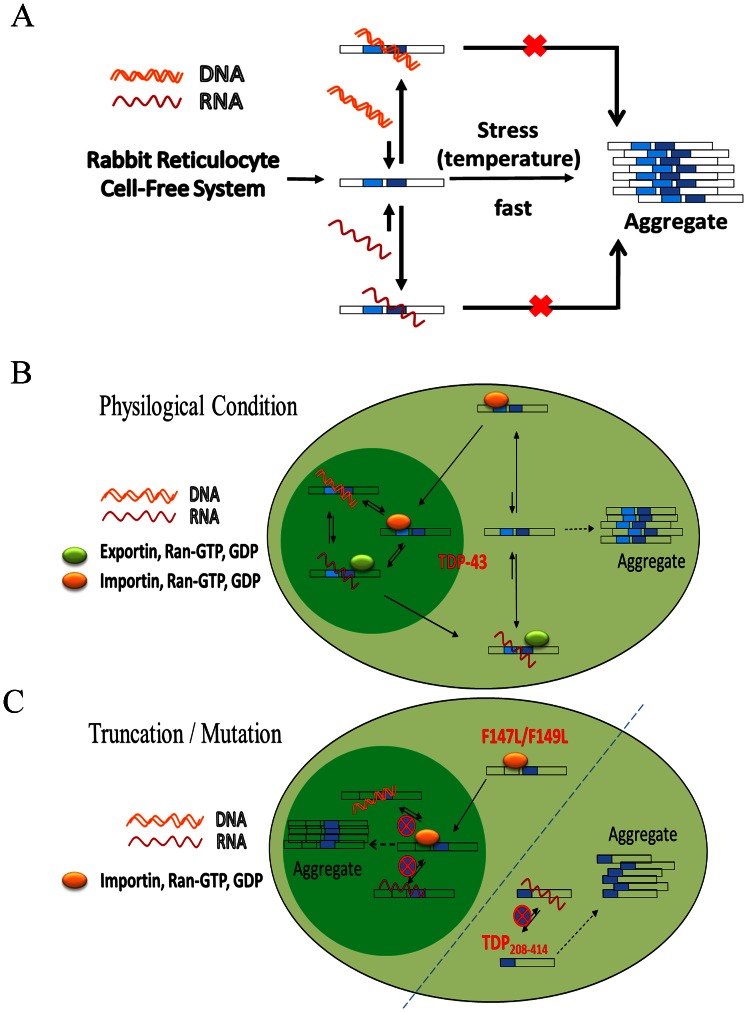
(A) The newly synthesized TDP-43 tends to aggregate when exposed to increased temperature (stress) in the rabbit reticulocyte cell free system. This propensity is highly suppressed when it’s bound to its cognate DNA/RNA. (**B**) The proposed model of TDP-43 and its interacting partners within the *Neuro2a* cell. The cognate DNA/RNA may shift the equilibrium of TDP-43 toward its soluble form in the cytoplasm. (**C**) The mutated/truncated TDP-43 protein is failed to interact with DNA/RNA and result in the aggregation in the nucleus and the cytosol, respectively.

Aggregation of TDP-43 has long been recognized as a key step in ALS and FTLD though the detailed mechanism remains vaguely understood. Till now, the reported factors that directly/indirectly influence the TDP-43 aggregation process include environmental temperature, oxidative stress, formation of stress granules, phosphorylation of C-terminus, the presence of C-terminal fragments, and the presence of N-terminal domain [4], [10], [15], [25], [45], [46]. Here, we add to the factor list a new one, the binding of DNA/RNA, with a set of experimental evidences using *in vitro* and cell models. As shown in our rabbit reticulocyte cell-free system, the expressed TDP-43 readily transformed into an aggregated form upon exposure to stressor such as an increase in temperature. However, this process was largely retarded or prohibited in the presence of its cognate DNA/RNA (Figure 6A). In fact, other *in vitro* experiments had shown that expressed full-length TDP-43 was aggregation prone and able to form fibrillar aggregates, on the other hand, even the over expressed full-length TDP-43 remains in the soluble state within the cell. We thus propose that the solubility and function of TDP-43 is maintained by its binding partner, such us DNA and/or RNA, once synthesized from the ribosome and transported into the nucleus (Figure 6B). On the contrary, the loss of binding ability to DNA/RNA causes full-length TDP-43 to form large nuclear body as shown with the mutant TDP-43 (F147L/F149L) in the *Neuro2a* cell (Figure 6C). Similarly, the solubility of the TDP-43 in the cytoplasm is also influenced by its cognate RNA as proven from the aggregation of truncated protein (TDP_208–414_) which lacks NLS sequence and RNA recognition motifs (Figure 6C).

### Enhancement in Protein Solubility/folding upon DNA/RNA-protein Recognition

As pointed out from the previous literature, the intrinsically disordered proteins/domain may transform into ordered structure or start folding upon binding to its cognate RNA [47]. It has also been shown that the refolding rate of Arc repressor dimer may be accelerated by 30 folds or more by the electrostatic interactions with single stranded or double stranded DNA/RNA [48]. Moreover, Choi *et. al.* had shown that the fusion of an RNA-binding module in an aggregation-prone protein may promotes the solubility of the passenger protein upon binding with RNA and proposed that RNA binding may influence the kinetic network of protein folding pathway toward productive folding over off-pathway aggregation [47]. In fact, the TDP-43 used in our studies is also a DNA/RNA binding protein and containing a C*-*terminus with large percentage of intrinsically disorder regions as predicted by the Predictors of Natural Disordered Regions (PONDR) [46]. According to our solubility studies in the rabbit reticulocyte cell-free system and the turbidity assay with the purified TDP-43, we similarly observed that the solubility of the TDP-43 was largely increased upon interacting with its cognate DNA/RNA. Though we are not yet sure whether the prevention of TDP-43 aggregation is resulted from the structural transformation in the disordered region or from the induced folding, it is obvious that the binding with DNA/RNA maintains the TDP-43 in its soluble form.

### The Solubility of TDP-43 under Normal and Pathological Conditions

TDP-43 may cooperate with many partners (e.g. RNA, DNA, exportin, importin, hnRNP) to participate in mRNA splicing, degradation, stabilization, translation and transportation within cells. We had shown that the cognate ssDNA and ssRNA rather than the hnRNP may directly regulate the solubility of TDP-43 and proposed that the binding partners/collaborators might help to diminish the intrinsically aggregation-prone tendency in TDP-43. Recently, it was also shown that the oxidative stress promoted TDP-43 cross-linking via cysteine oxidation near the RRM2 domain and induced cytoplasmic stress granules (SGs). Similarly, they have shown that stress-induced cysteine cross-linking impaired TDP-43 splicing function that could be due to the abrogation of the RNA binding ability in TDP-43. This would functionally mimic the RNA-binding deficient mutants [49]. By comparing the cross-linked TDP-43 in normal and FTLD-TDP human brain tissue, it is shown that pathological TDP-43 aggregates are associated with increased disulfide bond formation. Previous studies proposed that the intimate interaction between pathological TDP-43 and RNA in the stress granules represented the early stage of aggregation formation happening in the neuronal and glial cells [50]. Recently, the participation of nucleic acids in affecting aggregation of other proteins involved in protein misfolding diseases has also been discussed in many articles and reviews [34], [35], [37], [38]. It is shown that the cellular prion protein (PrP^C^) may interact with nucleic acids *in vitro* and is transformed into a β-sheet rich structure similar to its disease-causing isoform (PrP^SC^). Moreover, the interaction between PrP and nucleic acid may result in the partial unfolding of the prion protein and the formation of amyloid-like structure [33], [35], [36]. In the case of Parkinson’s disease, while the double-stranded oligos can promote the aggregation in alpha-Synuclein, single-strand circular DNA or supercoiled plasmid DNA may protect the protein from fibrillation [37]. On the other hand, the cognate DNA may interact with the tumor suppressor protein (p53) and prevents its aggregation, which is similar to our case in TDP-43 [39], [40]. Though the specific mechanism of how TDP-43 loses its DNA or RNA binding ability in ALS and FLTU is not disclosed, it is likely that the cognate DNA or RNA are closely correlated with the solubility and stability of TDP-43 under the pathological conditions.

## Supporting Information

Figure S1 **The solubility of human TDP-43 expressed in** ***E. coli*** **cell-free system.** (**A**) The western blotting signals of TDP-43 generated at various temperatures in the supernatant (S) and pellet (P) fractions. (**B**) The solubility test of generated TDP-43 in the presence and absence of DNA under the incubation at either 37 or 24°C.
(DOC)

Figure S2 **Expression of human TDP-43 protein in the rabbit reticulocyte cell-free system (TNT Quick Coupled Transcription/Translation Systems).** (**A**) The reactions were analyzed by western blotting with anti-FLAG antibody in the absence (lane 1) and presence (lane 2) of the plasmid encoding for the FLAG-tagged TDP-43. Proteins in the cell-free reaction were stained by either ponceau S or coomassie blue to confirm a similar amount of the samples were analyzed. (**B**) Time course (1–4 h) of the FLAG-tagged TDP-43 expression in the cell-free system at 30°C. After 2 h, the protein synthesis was completed. The production of TDP-43 was quantified by western blotting and analyzed by Image J.
(DOC)

Figure S3 **Quantification of FLAG-TDP-43 protein in the rabbit reticulocyte cell-free system (TNT Quick Coupled Transcription/Translation Systems).** (**A**) Different concentrations of purified FLAG-trigger factor protein were loaded on the SDS-PAGE as a standard to quantify the expressed FLAG-TDP-43 in the cell-free system. (**B**) The TDP-43 protein concentration of the *E.coli* cell-free system is diluted at five times and has been used to compare the TDP-43 protein expression in the Rabbit cell-free system. All proteins were analyzed by western blotting with anti-Flag antibody.
(DOC)

Figure S4 **DNA binding assay for refolded TDP-43 proteins.** 0.1 µM FAM-labeled (TG)_12_ (*left*) and (CA)_12_ (*right)* were mixed with TDP-43 FL (wild type and mutant) at indicated concentrations. The mixtures were filtered through a nitrocellulose membrane, and ssDNA/protein complex trapped on the membranes were probed with florescence dye. The image was obtained by Typhoon TRIO variable mode imager (GE Healthcare).
(DOC)

Figure S5 **EM images of refolded TDP-43 in the absence and presence of single-strand DNA.** Both (**A**) small and (**C**) large aggregates are found in the refolded TDP-43 samples. Similar aggregates are found in the refolded TDP-43 in the presence of single-strand DNA, (TG)_12_, as shown in (**B**) and (**D**). The red arrows indicate the gold nanoparticles for immunogold staining. The small bars indicate 100 nm in (**A**), (**B**) and (**C**), and 500 nm in (**D**).
(DOC)

Methods S1 **Supporting methods.**
(DOC)
