# Supplementary material for: Inhibition of TDP-43 Aggregation by Nucleic Acid Binding
Source: PLoS One. 2013 May 30;8(5):e64002. doi: 10.1371/journal.pone.0064002 (PMC3667863; doi:10.1371/journal.pone.0064002)
Supplement: Figure S1 — The solubility of human TDP-43 expressed in E. coli cell-free system. (A) The western blotting signals of TDP-43 generated at various temperatures in the supernatant (S) and pellet (P) fractions. (B) The solubility test of generated TDP-43 in the presence and absence of DNA under the incubation at either 37 or 24°C. (DOC) [file pone.0064002.s001.doc]

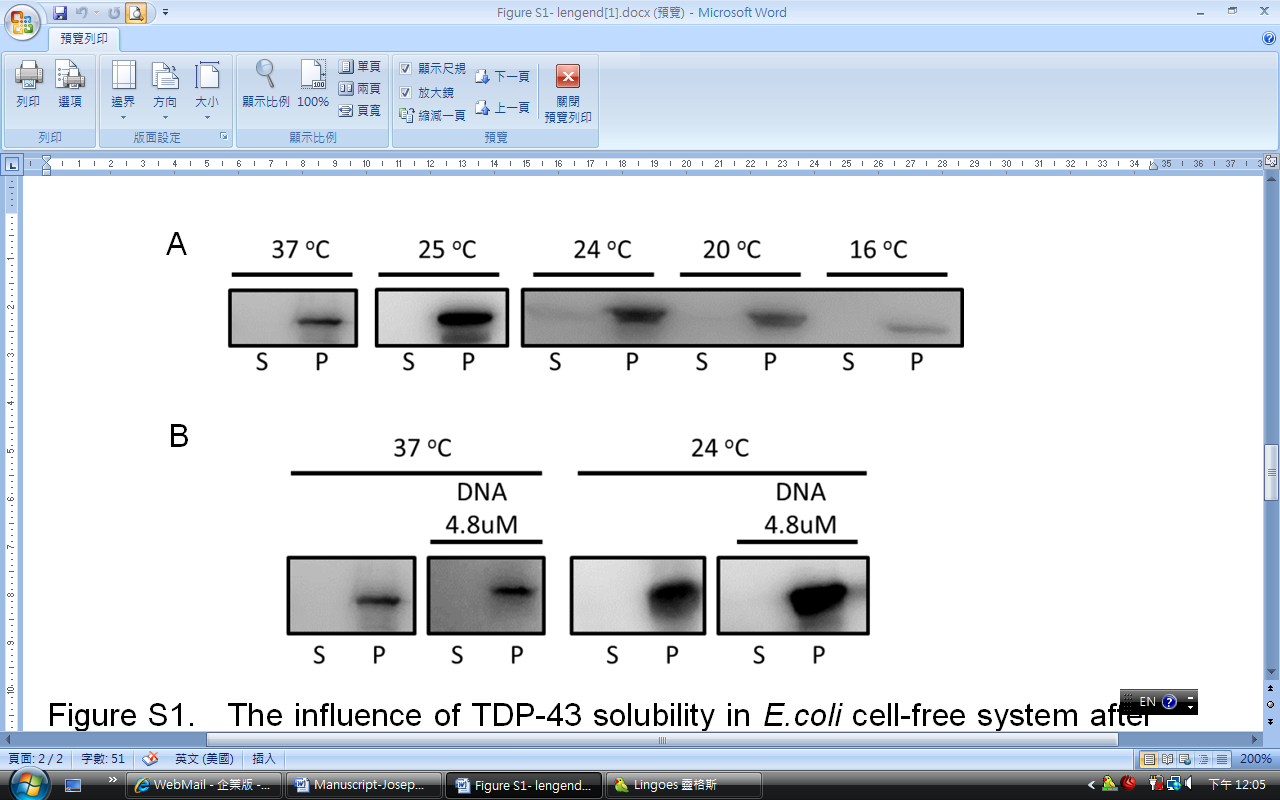


**Figure S1.** The solubility of human TDP-43 expressed in *E. coli* cell-free system. (**A**) The western blotting signals of TDP-43 generated at various temperatures in the supernatant (S) and pellet (P) fractions. (**B**) The solubility test of generated TDP-43 in the presence and absence of DNA under the incubation at either 37 or 24^o^C.
