# Supplementary material for: Inhibition of TDP-43 Aggregation by Nucleic Acid Binding
Source: PLoS One. 2013 May 30;8(5):e64002. doi: 10.1371/journal.pone.0064002 (PMC3667863; doi:10.1371/journal.pone.0064002)
Supplement: Figure S2 — Expression of human TDP-43 protein in the rabbit reticulocyte cell-free system (TNT Quick Coupled Transcription/Translation Systems). (A) The reactions were analyzed by western blotting with anti-FLAG antibody in the absence (lane 1) and presence (lane 2) of the plasmid encoding for the FLAG-tagged TDP-43. Proteins in the cell-free reaction were stained by either ponceau S or coomassie blue to confirm a similar amount of the samples were analyzed. (B) Time course (1–4 h) of the FLAG-tagged TDP-43 expression in the cell-free system at 30°C. After 2 h, the protein synthesis was completed. The production of TDP-43 was quantified by western blotting and analyzed by Image J. (DOC) [file pone.0064002.s002.doc]

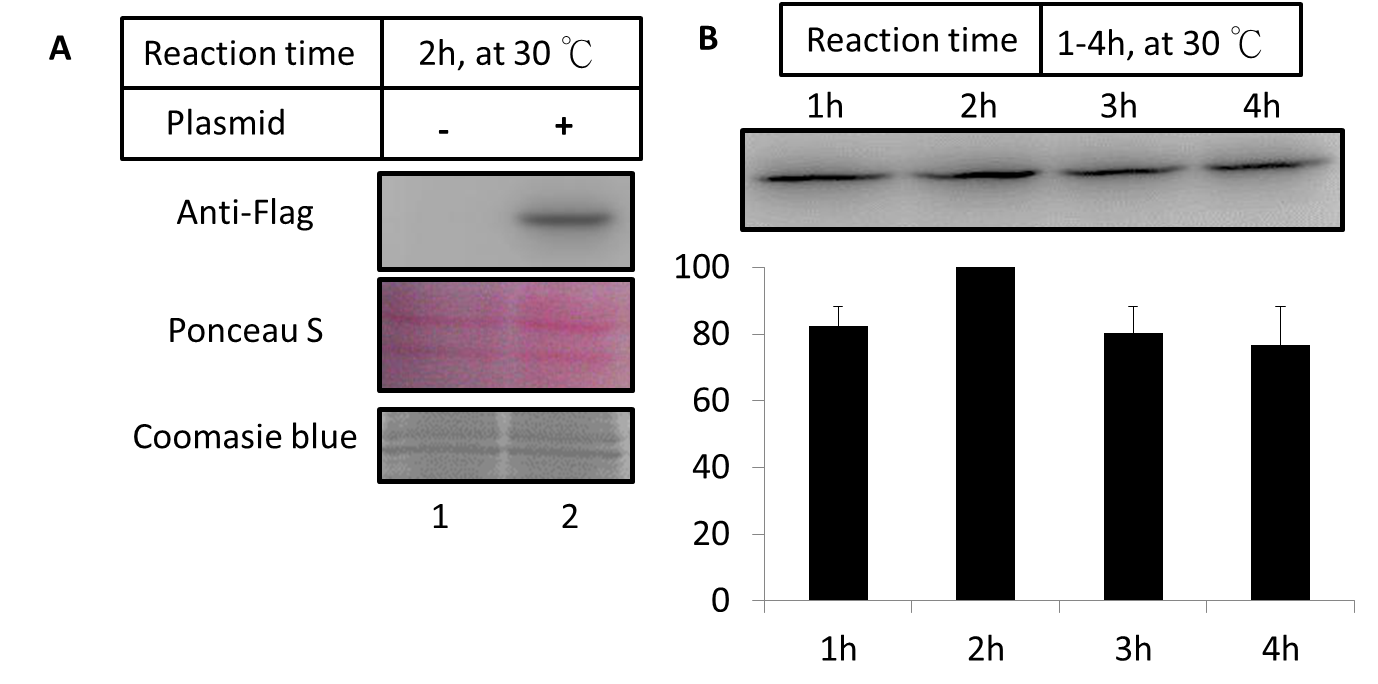


**Figure S2.** Expression of human TDP-43 protein in the rabbit reticulocyte cell-free system (TNT Quick Coupled Transcription/Translation Systems). (**A**) The reactions were analyzed by western blotting with anti-FLAG antibody in the absence (lane 1) and presence (lane 2) of the plasmid encoding for the FLAG-tagged TDP-43. Proteins in the cell-free reaction were stained by either ponceau S or coomasie blue to confirm a similar amount of the samples were analyzed. (**B**) Time course (1-4 h) of the FLAG-tagged TDP-43 expression in the cell-free system at 30^o^C. After 2 h, the protein synthesis was completed. The production of TDP-43 was quantified by western blotting and analyzed by Image J.
