# Supplementary material for: Inhibition of TDP-43 Aggregation by Nucleic Acid Binding
Source: PLoS One. 2013 May 30;8(5):e64002. doi: 10.1371/journal.pone.0064002 (PMC3667863; doi:10.1371/journal.pone.0064002)
Supplement: Figure S3 — Quantification of FLAG-TDP-43 protein in the rabbit reticulocyte cell-free system (TNT Quick Coupled Transcription/Translation Systems). (A) Different concentrations of purified FLAG-trigger factor protein were loaded on the SDS-PAGE as a standard to quantify the expressed FLAG-TDP-43 in the cell-free system. (B) The TDP-43 protein concentration of the E.coli cell-free system is diluted at five times and has been used to compare the TDP-43 protein expression in the Rabbit cell-free system. All proteins were analyzed by western blotting with anti-Flag antibody. (DOC) [file pone.0064002.s003.doc]

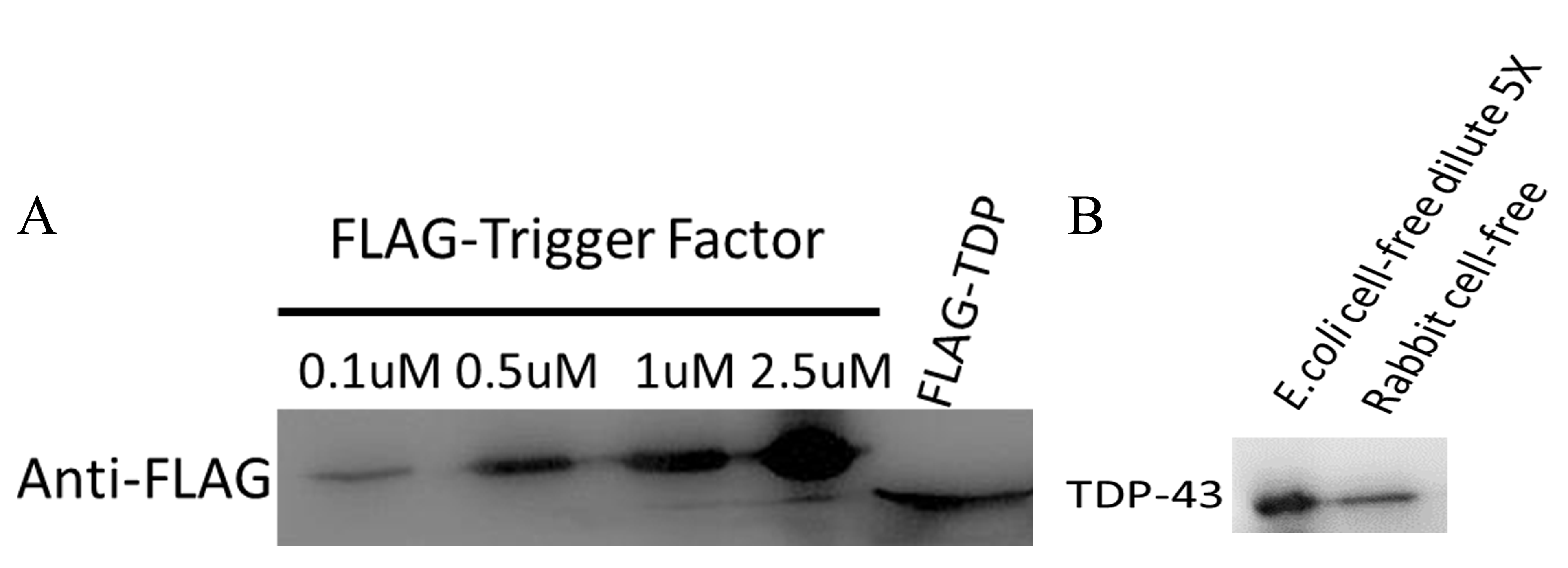


**Figure S3.** Quantification of FLAG-TDP-43 protein in the rabbit reticulocyte cell-free system (TNT Quick Coupled Transcription/Translation Systems). (**A**) Different concentrations of purified FLAG-trigger factor protein were loaded on the SDS-PAGE as a standard to quantify the expressed FLAG-TDP-43 in the cell-free system. (**B**) The TDP-43 protein concentration of the *E.coli* cell-free system is diluted at five times and has been used to compare the TDP-43 protein expression in the Rabbit cell-free system. All proteins were analyzed by western blotting with anti-Flag antibody.
