# Supplementary material for: Inhibition of TDP-43 Aggregation by Nucleic Acid Binding
Source: PLoS One. 2013 May 30;8(5):e64002. doi: 10.1371/journal.pone.0064002 (PMC3667863; doi:10.1371/journal.pone.0064002)
Supplement: Figure S4 — DNA binding assay for refolded TDP-43 proteins. 0.1 µM FAM-labeled (TG)12 (left) and (CA)12 (right) were mixed with TDP-43 FL (wild type and mutant) at indicated concentrations. The mixtures were filtered through a nitrocellulose membrane, and ssDNA/protein complex trapped on the membranes were probed with florescence dye. The image was obtained by Typhoon TRIO variable mode imager (GE Healthcare). (DOC) [file pone.0064002.s004.doc]

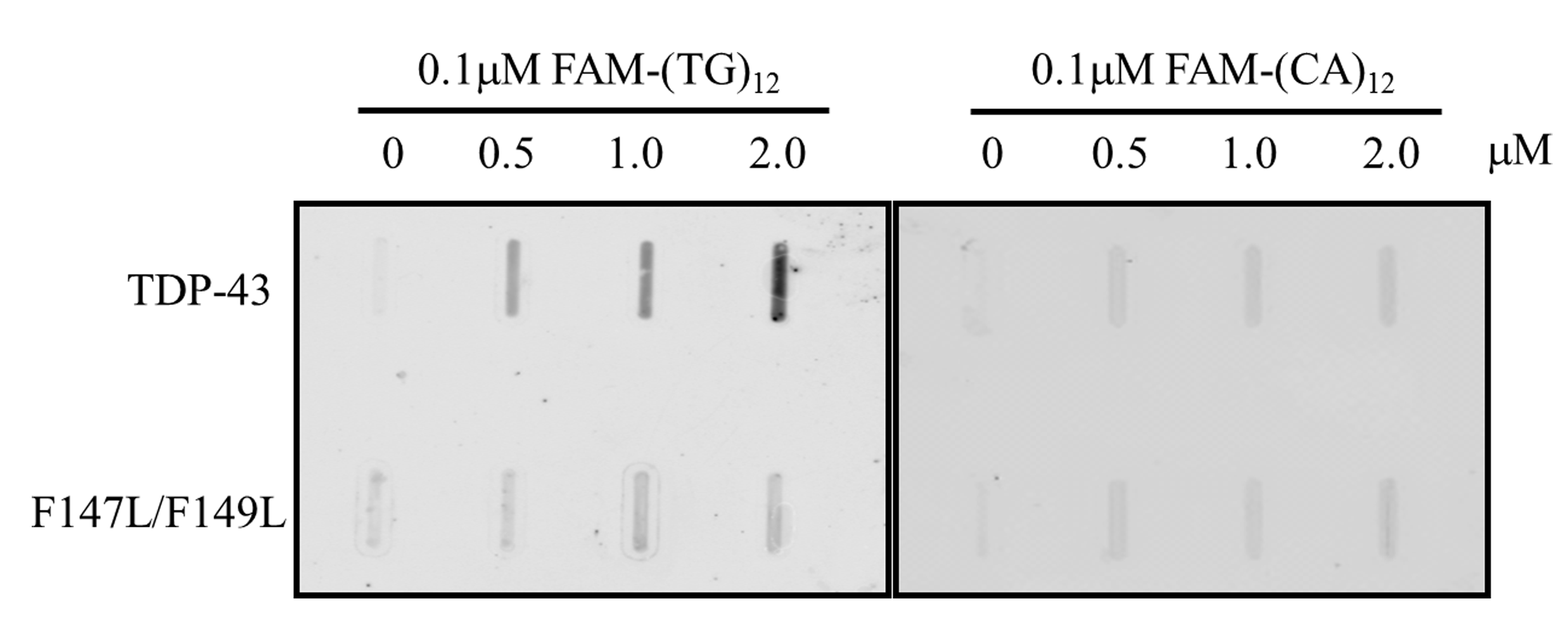


**Figure S4.** DNA binding assay for refolded TDP-43 proteins. 0.1 μM FAM-labeled (TG)_12_ (*left*) and (CA)_12_ (*right)* were mixed with TDP-43 FL (wild type and mutant) at indicated concentrations. The mixtures were filtered through a nitrocellulose membrane, and ssDNA/protein complex trapped on the membranes were probed with florescence dye. The image was obtained by Typhoon TRIO variable mode imager (GE Healthcare).
