# Supplementary material for: Inhibition of TDP-43 Aggregation by Nucleic Acid Binding
Source: PLoS One. 2013 May 30;8(5):e64002. doi: 10.1371/journal.pone.0064002 (PMC3667863; doi:10.1371/journal.pone.0064002)
Supplement: Figure S5 — EM images of refolded TDP-43 in the absence and presence of single-strand DNA. Both (A) small and (C) large aggregates are found in the refolded TDP-43 samples. Similar aggregates are found in the refolded TDP-43 in the presence of single-strand DNA, (TG)12, as shown in (B) and (D). The red arrows indicate the gold nanoparticles for immunogold staining. The small bars indicate 100 nm in (A), (B) and (C), and 500 nm in (D). (DOC) [file pone.0064002.s005.doc]

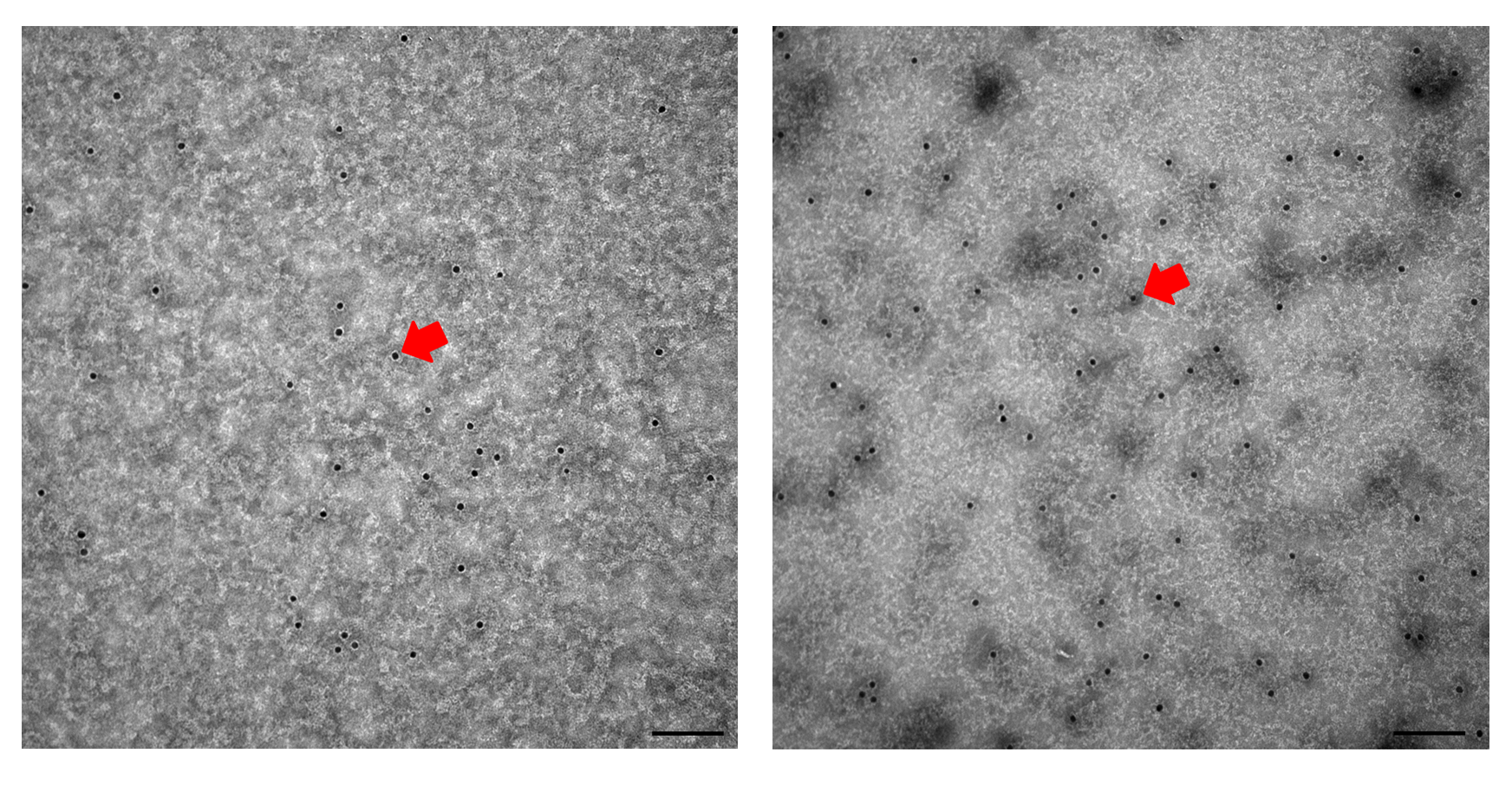


**A**

**C**

**B**

**D**

**
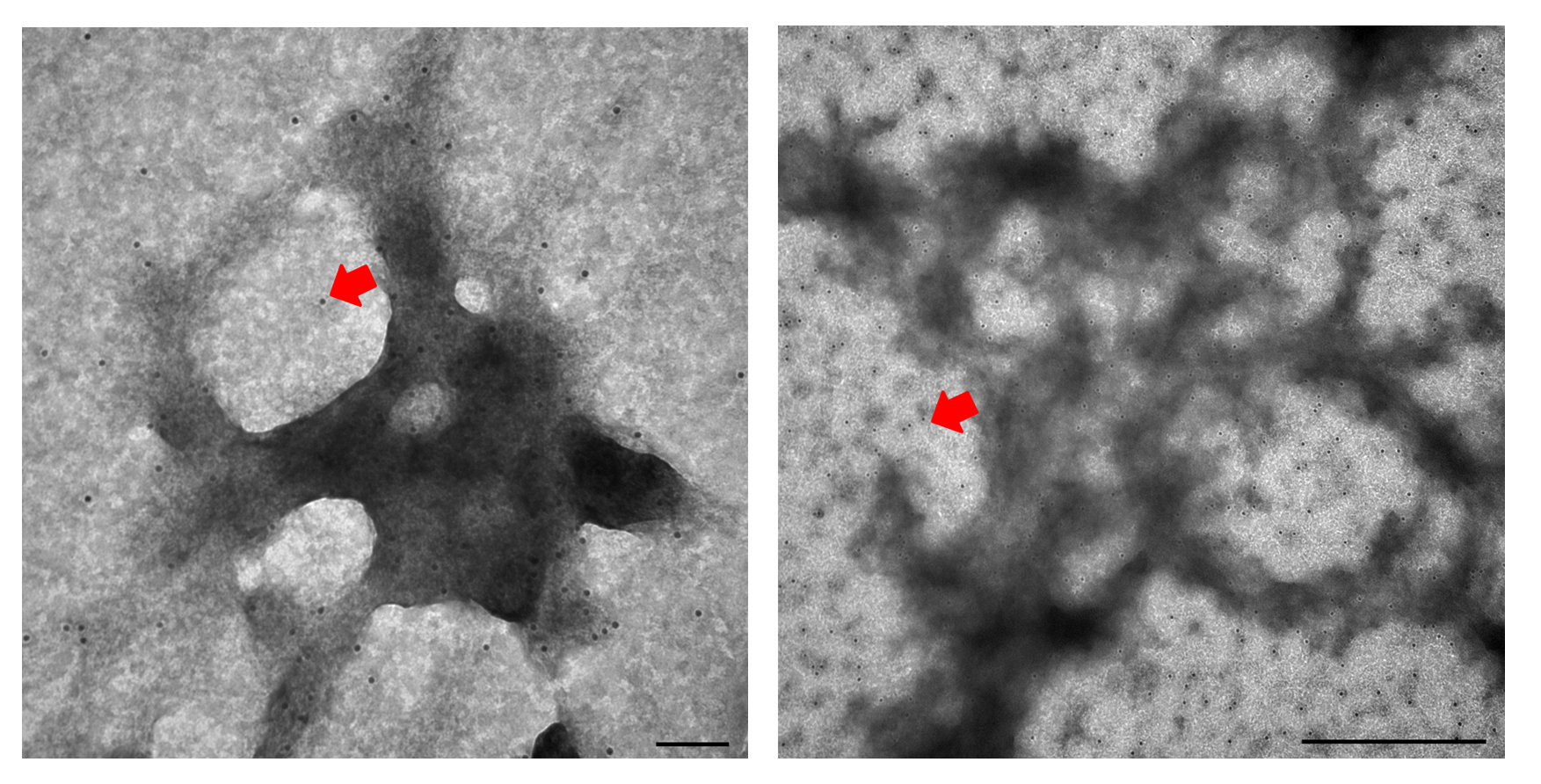
**

**Figure S5*.*** EM images of refolded TDP-43 in the absence and presence of single-strand DNA. Both (**A**) small and (**C**) large aggregates are found in the refolded TDP-43 samples. Similar aggregates are found in the refolded TDP-43 in the presence of single-strand DNA, (TG)_12_, as shown in (**B**) and (**D**). The red arrows indicate the gold nanoparticles for immunogold staining. The small bars indicate 100 nm in (**A**), (**B**) and (**C**), and 500 nm in (**D**).
