# Supplementary material for: Inhibition of TDP-43 Aggregation by Nucleic Acid Binding
Source: PLoS One. 2013 May 30;8(5):e64002. doi: 10.1371/journal.pone.0064002 (PMC3667863; doi:10.1371/journal.pone.0064002)
Supplement: Methods S1 — Supporting methods. (DOC) [file pone.0064002.s006.doc]

**Supplementary Methods**

**Expression and purification of TDP-43 and hnRNP A2 proteins.** To express the TDP-43 proteins, the pET17b (+)-TDP-43 and -TDP43-F147L/F149L constructs possessing a N-terminal His-tag were transformed into *E. coli* BL21 (DE3) and grown in the LB medium with ampicillin (100 μg/ml) at 30^o^C overnight without any chemical induction. The cells were harvested by centrifugation at 8,000 × *g* when the optical density at 600 nm reached or was higher than 1, and a homogeneous lysate in the PB buffer (10 mM phosphate, 35 mM NaCl, pH 8.0) was then obtained with French Press at 20,000 psi. The inclusion bodies in the pellet fraction of the lysate were collected and then dissolved in the denaturing buffer (PB buffer with 6 M urea and 0.2 mM EGTA). The denatured, soluble proteins were purified by Ni^2+^-affinity column (GE Healthcare) chromatography and the TDP-43 proteins bound to the Ni^2+^ column were eluted by elution buffers (100-200 mM imidazole in denaturing buffer) and then refolded by dialysis in the refolding buffer (10 mM phosphate, 1 mM EDTA, pH 8.0). After dialysis, soluble TDP-43 was further collected by centrifugation at 15,000 × *g* for 30 min in the supernatant fraction and concentrated by Amicon Ultra (Millipore) if necessary. The purified TDP-43 protein was characterized by western blotting with a rabbit *anti*-TDP-43-tag pAb (Genscript) and secondary anti-rabbit antibody conjugated with HRP. The protein quantification was performed by the Bradford method (Sigma-Aldrich) using bovine serum albumin (BSA, Sigma-Aldrich) as a standard. To express hnRNP A2 protein, the pET23a-hnRNP A2 construct containing N-terminal His-tag was transformed into *E. coli* BL21 (DE3) and grown in the LB medium with ampicillin (100 μg/ml) at 37^o^C. IPTG was added when the optical density (600 nm) reached 0.5. The cell was harvested by centrifugation at 8,000 × *g* and a homogeneous lysate in the Hepes buffer (20mM Hepes-Na, 1mM EDTA, 5% glycerol, 1mM DTT, 1mM PMSF, pH 8.0) was then obtained with French Press at 20,000 psi after 4h. The supernatant fraction was collected and were purified by Ni^2+^-affinity column (GE Healthcare) chromatography and the TDP-43 proteins bound to the Ni^2+^ column were eluted by elution buffers (200 mM imidazole in the Hepes buffer).

**Immunofluorescene staining.** *Neuro2a* cells were transfected with vectors pCMVTNT-FLAG-TDP43, pCMVTNT FLAG-TDP43-F147L/F149L, or pCMVTNT-FLAG-TDP43_208-414_ and incubated at 37°C for 36 h in DMEM with 10% FBS. The cells were fixed in 4% (v/v) paraformaldehyde in PBS (137 mM NaCl, 2.7 mM KCl, 10 mM Na_2_HPO_4_, 1.8 mM KH_2_PO_4_, pH 7.4) for 15 minutes at 4°C followed by treatment with 0.2% (v/v) Triton X-100 (Sigma, USA) in PBS for 10 minutes and incubation in 2% (w/v) BSA in PBS for 20 minutes. The TDP-43 proteins within the cells were hybridized with anti-FLAG M2 antibodies by incubation at 25 °C for 2 h followed by incubation with a secondary anti-mouse antibody that conjugated with Alexa Fluor 488 (kindly provided by Dr. Benjamin Tu, Academia Sinica). In addition, the cell nuclei were stained by 0.01% (w/v) 4', 6-diamidino-2-phenylindol (DAPI) in PBS at room temperature for 10 minutes. Microscope slides were mounted with 50% glycerol in PBS and sealed with nail polish. Fluorescence was detected by Zeiss LSM 510 META/NLO Confocal Laser Scanning Microscope (Carl Zeiss AG, Germany).
